# 12 years of assembly patterns in saproxylic beetles suggest early decay wood as ephemeral resource patch

**DOI:** 10.1111/1365-2656.70183

**Published:** 2025-11-11

**Authors:** Ludwig Lettenmaier, Claus Bässler, Orsi Decker, Jonas Hagge, Christoph Heibl, Giorgi Mamadashvili, Sebastian Seibold, Simon Thorn, Jörg Müller

**Affiliations:** ^1^ Field Station Fabrikschleichach, Chair of Conservation Biology and Forest Ecology, Biocenter University of Würzburg Rauhenebrach Germany; ^2^ Bavarian Forest National Park Grafenau Germany; ^3^ Fungal Ecology, Bayreuth Center of Ecology and Environmental Research (BayCEER) University of Bayreuth Bayreuth Germany; ^4^ Department of Forest Nature Conservation Northwest German Forest Research Institute Hann. Münden Germany; ^5^ Department of Forest Nature Conservation University of Göttingen Göttingen Germany; ^6^ Forest Zoology TUD Dresden University of Technology Tharandt Germany; ^7^ Hessian Agency for Nature Conservation, Environment and Geology State Institute for the Protection of Birds Gießen Germany; ^8^ Applied Ecology Philipps Universität Marburg Marburg Germany; ^9^ Biology Centre, Institute of Entomology Czech Academy of Sciences České Budějovice Czech Republic

**Keywords:** biodiversity, coleoptera, habitat filtering, habitat‐heterogeneity hypothesis, more‐individuals hypothesis, traits

## Abstract

1. The ephemeral resource patch (ERP) concept provides a framework for understanding how finite, short‐lived resources shape community assembly processes at both patch and landscape scale. Some of these theories and principles can be applied to intermediate‐lived resources, such as deadwood, but this remains largely unexplored. We tested three ecological mechanisms of community assembly (*more‐individuals hypothesis*, *habitat‐heterogeneity hypothesis* and *habitat filtering*) to investigate whether beetle assemblages in deadwood fit the ERP concept.
2. We tracked saproxylic beetle communities in experimental logs of Norway spruce (*Picea abies*), European silver fir (*Abies alba*) and beech (*Fagus sylvatica*) in the temperate mountain forest of the Bavarian Forest National Park over a 12‐year decomposition period, from the early decomposition stage until near‐complete resource depletion.
3. Beetle abundance and number of species declined consistently in all tree species until the 4th year but increased again in spruce after ~8 years. Species richness (number of species controlled for abundance) showed inconsistent patterns over time: U‐shaped for spruce, weakly hump‐shaped for fir and no temporal effect for beech. Habitat filtering was more pronounced in the early stage as functional diversity was initially low but increased for all tree species up to 4 years, then plateaued and increased again after ~10 years for both conifers. Conditional inference tree identified two temporally distinct beetle assemblages (years 1–3 and 4–12), and strong differences within the first 4 years.
4. Our findings suggest that the *more‐individuals hypothesis* and *habitat filtering* are key mechanisms driving community assembly in saproxylic beetles. Early decomposition stages supported functionally similar assemblages, highlighting this phase as a critical period for decomposer community structuring.
5. *Synthesis*. The consistency of the early successional trajectories of beetles suggests that the early stages of deadwood decomposition up to the 3rd year in the temperate zone follow ephemerality theories similar to those of short‐lived ERPs, while the advanced stages provide a habitat for a more random combination of beetle species. Furthermore, our findings highlight the need for temporally continuous deadwood input, via natural processes or staggered retention during logging operations, to provide coarse woody debris for wide range of saproxylic beetles.

## Graphical abstract

We reveal that early stages of deadwood decomposition follow ecological rules of ephemeral resource patches. By tracking beetle communities over 12 years, we show how decomposition dynamics shape community assembly patterns, highlighting the importance of continuous deadwood input for sustaining saproxylic beetles in temperate forests.

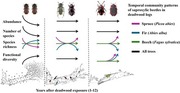

## 1 INTRODUCTION

Resource availability plays a fundamental role in shaping the dynamics of animal communities (Elton, 1949; Nyman, 2010). In particular, finite resources and their stochasticity can shape entire ecosystems by driving source‐sink dynamics (Amarasekare & Nisbet, 2001), intensifying competition (Rohlfs & Hoffmeister, 2004) or promoting species coexistence (Germain et al., 2021). Finite resources range from short‐lived ephemeral sources such as fungal fruiting bodies, leaf litter, animal dung and carrion to intermediate‐lived resources such as decaying coarse woody debris and long‐lived resources such as ephemeral rivers (Benbow et al., 2019; Butterworth et al., 2022). Benbow et al. (2019) and Butterworth et al. (2022) synthesized research on finite resources. The latter introduced the concept of *ephemeral resource patches* (ERPs), focusing on theories and principles mainly applicable to short‐lived resources, but they can also be applied to intermediate‐lived resources.

ERPs vary at two spatial scales: the patch scale (local) and the landscape scale (metapopulation), both of which influence eco‐evolutionary processes in decomposer communities. Butterworth et al. (2022) identified four patch‐scale characteristics: (1) volume and shape: the spatial dimensions of the patch; (2) ephemerality: how long the resource persists and can be consumed; (3) community structure: the abundance, diversity and species identities of the patch community; (4) heterogeneity: the structural and chemical diversity of the patch and the number of distinct niches, which change over time. These characteristics should shape consumer behaviour, with phylogenetic constraints limiting adaptive response in dispersal, detection and resource use (see Butterworth et al., 2022 and references therein).

While landscape‐scale characteristics of deadwood are well known to influence the dispersal of deadwood inhabiting species and the dynamics of their metapopulations in line with many types of ERPs (Butterworth et al., 2022; Jonsson et al., 2005; Komonen & Müller, 2018; Moor et al., 2021; Seibold et al., 2017; Sverdrup‐Thygeson et al., 2014), there are still open questions on the role of patch‐scale characteristics of this intermediate‐longevity resource, compared to short‐lived ERPs. The effects of some patch‐scale characteristics—such as volume, shape and heterogeneity—on saproxylic insect communities are well understood: large volumes of deadwood are associated with high abundance, number of species and functional diversity (Gossner et al., 2013; Lassauce et al., 2011; Parisi et al., 2018). On the other hand, resource heterogeneity within deadwood drives species composition and diversity (Lettenmaier et al., 2022; Müller et al., 2020; Seibold et al., 2016). Although it is known that the energy content and chemical composition of deadwood change over time (ephemerality) (Filipiak, 2018), and that distinct species assemblages use this resource over time (community structure) (Müller et al., 2020), the timing and mechanisms of these changes remain poorly understood—largely due to a lack of continuous long‐term studies. This gap is critical for forest management and conservation, as decomposition dynamics differ between necromass types (dead organic matter of any type) (Benbow et al., 2019; von Hoermann et al., 2023). To fully understand how ERPs shape ecological and evolutionary dynamics, Butterworth et al. (2022) argue for a contextual approach that integrates spatiotemporal metrics with phylogenetic and trait‐based constraints.

Three major mechanisms can help to link community assembly patterns and theories in ERPs in deadwood: (i) the *more‐individuals hypothesis*, (ii) *habitat filtering* and (iii) the *habitat‐heterogeneity hypothesis*. The *more‐individuals hypothesis* (Srivastava & Lawton, 1998), suggests that larger amounts of resources with more available energy (Clarke & Gaston, 2006) support more individuals and thus more species. In the early stage of decomposition, high‐energy nutrients in the inner bark may support high beetle abundance and thus the number of species (Filipiak, 2018; Stokland et al., 2012). *Habitat filtering* (Balmford, 1996) selects species based on trait‐environment matching (Götzenberger et al., 2012; Keddy, 1992), indicating species coexistence or competition. During early decomposition, selective pressure is high due to the short availability of high‐energy nutrients and the presence of plant defensive compounds (Filipiak, 2018), as well as the physical barrier of intact bark and wood, which requires colonizers to rely on earlier arrivals that bore entry holes (Hagge, Bässler, et al., 2019; Zuo, Berg, et al., 2016). Beetles colonizing at this stage are specialized to cope with these defences (Stokland et al., 2012; Ulyshen & Hanula, 2010; Wende et al., 2017). The *habitat‐heterogeneity hypothesis* (MacArthur, 1984) predicts that more diverse niches support more species independent of abundances (Seibold et al., 2016). In the advanced stage, fungal degradation of plant defences weakens habitat filtering and structural deadwood heterogeneity increases and thus niche availability. This allows for less clustered beetle assemblages (Stokland et al., 2012). In addition, early colonizers can alter biotic conditions, increasing habitat heterogeneity and ultimately influencing community patterns at advanced stages, also known as the priority effect (Chase, 2003; Fukami et al., 2010).

**FIGURE 1:**
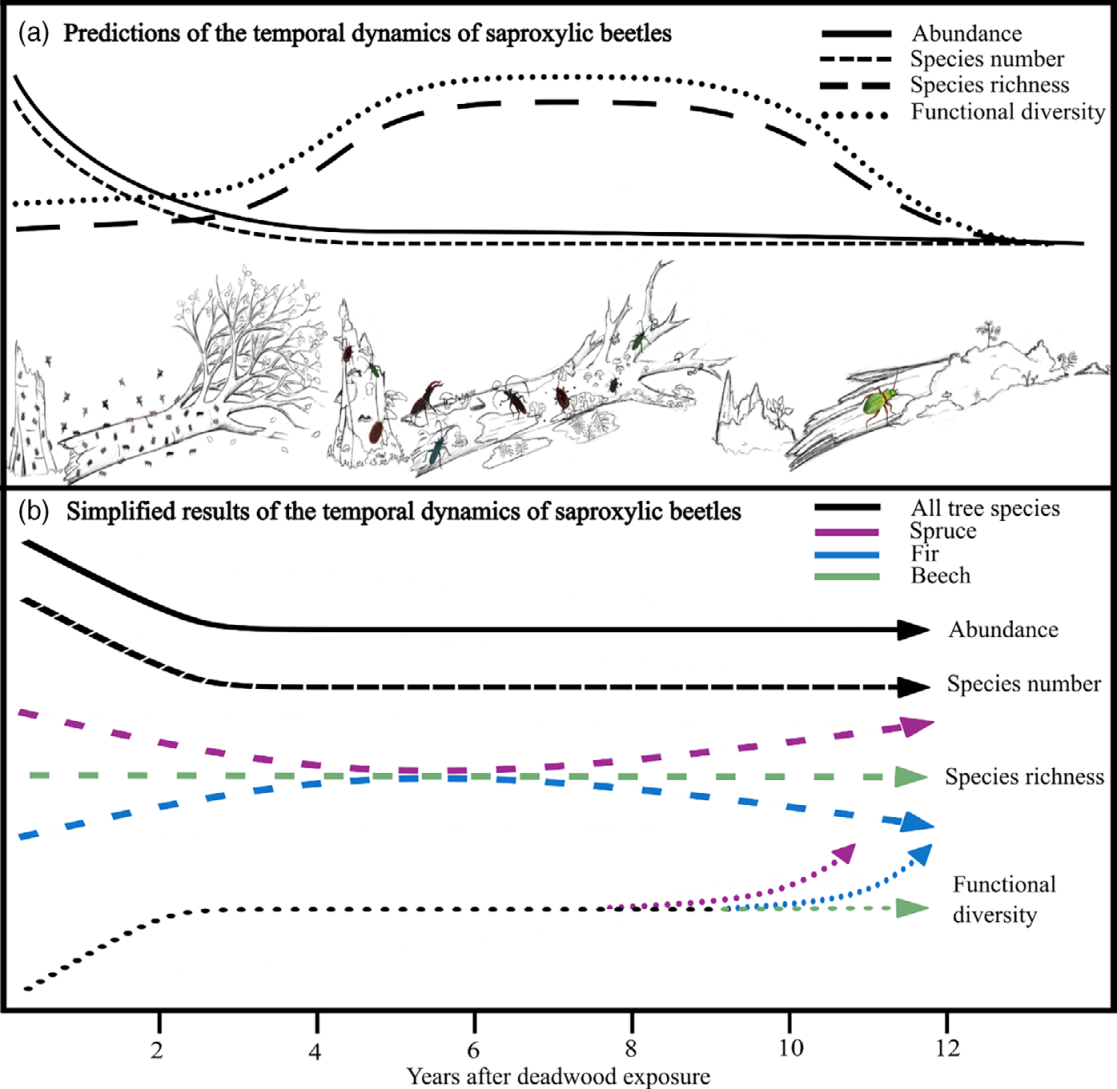
(a) Predicted changes in beetle assemblages during deadwood decomposition. Predictions include an early decline in beetle abundance and species number, but stable species richness (*more‐individuals hypothesis*) and low functional diversity (strong *habitat filtering*). In advanced decomposition, beetle species richness (*habitat‐heterogeneity hypothesis*) and functional diversity (weaker *habitat filtering*) are expected to increase. Predictions are based on known successional changes in nutrient compounds (Filipiak, 2018), fungal and microbial communities (Rajala et al., 2012, 2015), and structural heterogeneity (Přívětivý & Šamonil, 2021). (b) Simplified results from our 12‐year experiment, showing observed beetle dynamics of abundance, species number, species richness and functional diversity across spruce (*Picea abies*), fir (*Abies alba*) and beech (*Fagus sylvatica*). Reliability of stem emergence traps declined in the late decomposition stage due to advanced structural breakdown of logs, limiting sampling beyond year 12.

Saproxylic beetles are ideal study organisms for quantifying community characteristics in deadwood and the mechanisms involved within the ERP concept. They form a prominent multitrophic and co‐occurring group of deadwood colonizers that have repeatedly evolved adaptations to use deadwood across lineages (Hagge et al., 2021; Ulyshen, 2018). We collected saproxylic beetle assemblages annually for 12 years from experimental deadwood logs of the three dominant tree species (*Picea abies*, *Abies alba* and *Fagus sylvatica*) in the Bavarian Forest National Park, Germany. This timeframe allowed us to study the whole process of deadwood decomposition until the dissolution of the outer trunk (see Supporting Information Section 1: Figure S1). Specifically, we investigated abundance, observed number of species (species density; sensu Gotelli & Colwell, 2001), species richness (species number controlled for abundance; sensu Gotelli & Colwell, 2001), functional diversity (based on combined functional and phylogenetic information; Cadotte et al., 2013), niche breadth and community composition. We differentiate between species number and species richness following Gotelli and Colwell (2001). Species number (also referred to as species density) is defined as the observed number of species within a given sampling unit. This measure is influenced by the number of individuals collected, as more individuals lead to a higher chance of sampling more species (*more‐individuals hypothesis*). In contrast, species richness aims to account for this sampling effect by controlling for differences in abundance, thus providing a more comparable measure of diversity across samples with varying numbers of individuals. If the number of observed species can be simply explained by abundance, this supports the *more‐individuals hypothesis*. If we find more species than can be explained by abundance (i.e. species richness), this supports the *habitat‐heterogeneity hypothesis* (Seibold et al., 2016; Wierer et al., 2024). Specifically, for the early decomposition stage, we hypothesized (H1) a strong decline in abundance and number of species in response to high energy‐rich nutrient availability, but stable species richness (*more‐individuals hypothesis*). For the advanced decomposition stage, we expect (H2) lower abundance due to nutrient depletion, but higher species richness due to an increase in fungal species and structural heterogeneity of the deadwood (*habitat‐heterogeneity hypothesis*). We hypothesize (H3) that the early decomposition stage hosts low functional diversity due to strong *habitat filtering* driven by plant defensive compounds and nutrient availability. In contrast we hypothesize (H4) advanced decomposition stages to support higher functional diversity due to relaxed *habitat filtering* (Figure 1a). Overall, we expect (H5) distinct community compositions in the early decomposition stages compared to the advanced stages.

## 2 MATERIALS AND METHODS

### 2.1 Study area, experimental design and species sampling

We used data from saproxylic beetles sampled from 52 deadwood logs for 12 years belonging to two different deadwood experiments conducted in the Bavarian Forest National Park in south‐eastern Germany (Seibold et al., 2016; Thorn et al., 2016). The experimental plots for both studies were located within the management zone of the Bavarian Forest National Park. The forested area consists of broadleaf and coniferous trees, creating a mosaic of closed‐canopy forests interspersed with sunny gaps caused by bark beetle activity (Müller et al., 2010). The two experiments were conducted in forests with a similar structure and composition. Saproxylic beetles were sampled by using stem emergence traps fixed on the upper side of a deadwood log (covering ≈0.35 m^2^ of the stem). The advantage of stem emergence traps is that they are attached directly to the surface of the deadwood, allowing them to sample only organisms emerging from the wood, rather than capturing flying or crawling organisms outside the resource. In later decomposition stages we used additional metal rings to seal the stem emergence traps. Furthermore, the traps were inspected monthly to detect and repair any damage or detachment. The first experiment started in autumn 2011 by exposing freshly cut deadwood logs (diameter: ~30 cm, length: 5 m) on 30 plots. On each plot either European beech and/or silver fir logs were deposited, resulting in 10 beech, 10 fir and 10 mixed beech‐fir plots. Stem emergence traps were installed in spring, 2013. One stem emergence trap was affixed to one log of each tree species at each plot (i.e. one trap at single‐tree species plots and two at two‐tree species plots), resulting in a total of 40 traps. Beetles were collected monthly from May to August for 12 years. The second experiment started in April 2013 by uprooting Norway spruce trees on 12 plots. Sampling of spruce trees started in the same year (2013) and in the following 12 years, always from April to September. No sampling in both experiments was performed in the year 2020. Therefore, all logs were sampled from 2013 to 2019 and 2021 to 2024. Every year the emergence traps were moved to another part of the trunk to allow continuous colonization over time. Since spruce logs were exposed with a one‐year delay compared to beech and fir, we defined the sample year ‘2013’ for spruce as the ‘0’ year of deadwood exposure, while 2013 is the first year of deadwood exposure for beech and fir. After 12 years of exposure, most deadwood logs could no longer support the attachment of a stem emergence trap due to significant degradation (see Section 1 of Supporting Information Figure S1). For more details of the experimental setup and site description see Seibold et al. (2016) and Thorn et al. (2016) and Section 1 of Supporting Information. Our study did not require ethical approval.

### 2.2 Beetle traits and phylogeny

Beetles were identified to species level by taxonomic experts (Boris Büche and Alexander Szallies). We extracted information on morphological and ecological traits from identified beetle species responsible for important ecological functions from literature (Gossner et al., 2013; Hagge et al., 2021; Köhler, 2000; Seibold et al., 2015). In particular, we selected traits that are directly related to the colonization of deadwood and the survival and reproduction of beetles in deadwood (see Barton et al., 2011; Burner et al., 2021; Drag et al., 2023; Gossner et al., 2013, 2016; Hagge et al., 2021; Neff et al., 2022; Seibold et al., 2015; Stokland et al., 2012 and references therein). The final selection included 13 morphological traits (body length, body width, body roundness, head length, wing length, wing aspect, wing load, leg length, antenna length, eye length, hairiness, mandibular aspect and colour), some standardized by body length (Hagge et al., 2021) or log‐transformed (Supporting Information Section 2: Table S1). Additionally, we selected four ecological traits (decay niche, wood diameter niche, feeding type and host tree association) from literature (Gossner et al., 2013; Köhler, 2000; Seibold et al., 2015). There were strong correlations between body length and wing length (*r* = 0.66), body length and front femur length (*r* = 0.62), and wing length and front femur length (*r* = 0.63) (Section 2 of Supporting Information Figure S2). To assess multicollinearity among the morphological trait variables, we calculated Variance Inflation Factors (VIFs) using a linear model with a constant dummy response. Specifically, we fitted a linear model with all trait variables as predictors and a constant as the response, then extracted VIF values using the *car* package (Fox & Weisberg, 2018). All VIF values were below 5, indicating no problematic multicollinearity. For the phylogenetic information, we used a published phylogeny for central European saproxylic beetles (Hagge et al., 2021). Five species missing in this phylogeny were assigned to the tip next to the closest known relative (Section 3 of Supporting Information).

### 2.3 Statistical analyses

We performed all statistical analysis in R version 4.3.1 (www.r‐project.org). To test our hypotheses H1 and H2 we first modelled the abundance from each deadwood log summed up per year, using the *gam* function with a negative binomial distribution from the *mgcv* package (Wood, 2017). The model included a tree species‐specific smooth term for years after deadwood exposure (treated as numeric) (s(exposure, by = tree species)) allowing each tree species to have its own nonlinear temporal trend. We also included tree species as a fixed effect to account for overall differences in abundance among tree species, regardless of exposure. This fixed effect captures baseline (intercept‐level) differences between tree species, while the smooth terms model how beetle abundances change over time differently for each tree species. This approach allows us to separate temporal dynamics from constant differences between tree species. Furthermore, we included deadwood log coordinates as a smooth term (s(E, N, bs = ‘tp’)) to account for the repeated measurements and spatial autocorrelation (Müller et al., 2024). For the number of species (species density; sensu Gotelli & Colwell, 2001) model we applied the same model structure, but with the number of species as the response variable. To explicitly test whether species richness (number of species controlled for abundance; sensu Gotelli & Colwell, 2001) patterns were driven by differences in beetle abundance, we included log‐transformed abundance as an additional fixed effect and the number of species as the response variable (species richness model). This approach allowed us to test whether the observed number of species is driven by abundance alone (*more‐individuals hypothesis*). A variation in species richness across years after deadwood exposure, even after controlling for abundance, suggests that environmental factors, such as shifts in niche availability during decomposition or increasing habitat heterogeneity, are influencing beetle community structure (*habitat‐heterogeneity hypothesis*). For detailed model specifications, see Section 5 of Supporting Information. To assess potential spatial autocorrelation, we used the *testSpatialAutocorrelation* function from the *DHARMa* package, which calculates Moran's *I* for distance‐based spatial structure. No significant spatial autocorrelation was revealed (Section 5 of Supporting Information Table S4).

To assess habitat filtering on community assembly patterns (H3 and H4), we calculated mean functional‐phylogenetic distance (MFPD) following Cadotte et al. (2013). The key to MFPD is a weighting parameter *a*, which weights the contributions of functional and phylogenetic distances to MFPD. When *a* = 0, MFPD only includes functional distance and when *a* = 1, MFPD only includes phylogenetic distance. At intermediate values of *a*, both functional and phylogenetic distances contribute to MFPD. With this approach we account for unmeasured traits and for high phylogenetically conserved functional traits in beetles (Hagge et al., 2021). To calculate the functional distance matrix, we used Gower distance, which is able to account for categorical and continuous variables (Gower, 1971) with the function *daisy* from the *cluster* package (Maechler et al., 2022). The phylogenetic distance matrix was calculated by the function *cophenetic* from the *stats* package. To control for variation in the number of species in the samples and to obtain a metric on the assembly patterns affected by *habitat filtering*, we applied a null‐model approach to MFPD using the tip‐shuffling method (Cadotte & Davies, 2016). This provides standardized effect sizes (SES) of the mean pairwise functional‐phylogenetic distance (SES MFPD, referred to as ‘functional diversity’ for simplicity), which we calculated with 999 randomizations with the function *ses.mpd* in the package *picante* (Kembel et al., 2010). We fitted the same GAM model structure described above, but with functional diversity (SES MFPD) as the response variable and a Gaussian distribution. To determine the optimal balance between functional and phylogenetic contributions to the MFPD distance matrix, we re‐ran GAM models for a range of *a*‐values in intervals of 0.025. The final model was selected based on the highest adjusted *R* ^2^ value (*a* = 0.35, Section 4 of Supporting Information Figure S4). To assess the relative importance of individual traits in shaping functional diversity patterns, we conducted a trait‐exclusion analysis. Specifically, we iteratively removed one trait at a time and recalculated MFPD with *a* = 0.35. For each modified MFPD, we recomputed functional diversity with the previously described null model and re‐ran the corresponding GAM model. We then extracted the adjusted *R* ^2^ values of each model to evaluate how the removal of a single trait influenced explanatory power. We then compared the adjusted *R* ^2^ of each reduced model to that of the full model (adjusted *R* ^2^ = 0.243). A reduction in adjusted *R* ^2^ (Δ*R* ^2^) of ≥0.03 was interpreted as a substantial loss in explanatory power, indicating that the excluded trait strongly influenced functional diversity patterns (Section 4 of Supporting Information Table S2).

To test H5, we applied non‐metric multidimensional scaling (NMDS) using the *metaMDS* function in the *vegan* package, based on Bray–Curtis dissimilarity (Oksanen et al., 2023). This function performs a PCA‐like rotation of the NMDS solution axes which ensures that the first NMDS axis corresponds to the greatest variance among assemblages and allows further testing on the main variation of species composition (Müller et al., 2023; Seibold et al., 2016). To yield meaningful dissimilarity calculations, we included only deadwood logs that contained at least four beetle species within a given year. Assemblages from spruce logs sampled in year 0 (2013) were excluded, as fir and beech sampling started in the first year. To explore distinct community compositions between years of deadwood decomposition, the relationship between the scores of the first NMDS axis and years after deadwood exposure (treated as a factor to capture year‐specific effects) was modelled using a conditional inference tree (*ctree*) from the *partykit* package (Hothorn & Zeileis, 2015). Additionally, we applied a permutational multivariate analysis of variance (PERMANOVA) using the *adonis2* function with 999 permutations to assess the effects of tree species and years after exposure (treated as a factor to capture year‐specific effects) on beetle community composition.

To visualize the temporal emergence patterns of beetle species, we calculated the abundance‐weighted mean year of emergence for each beetle species, based on the number of individuals recorded per year. The standard deviation around this mean was used as a proxy for the temporal niche breadth. For visualization purposes, we only included those beetle species with at least three individuals in a given year, regardless of the deadwood log from which they emerged. All beetle species, including rare ones, are displayed in Figure S6 in Section 7 of Supporting Information.

## 3 RESULTS

Over 12 years of deadwood decomposition, we collected 35,059 individuals of 297 saproxylic beetle species from 46 families. Spruce, fir and beech logs contained 155 (15,271 individuals), 198 (11,506 individuals) and 219 species (8282 individuals), respectively. All three tree species had 98 species in common. Beech and fir shared 152 species, fir and spruce shared 113 species, and beech and spruce shared 108 beetle species (Section 8 of Supporting Information Figure S8).

**FIGURE 2:**
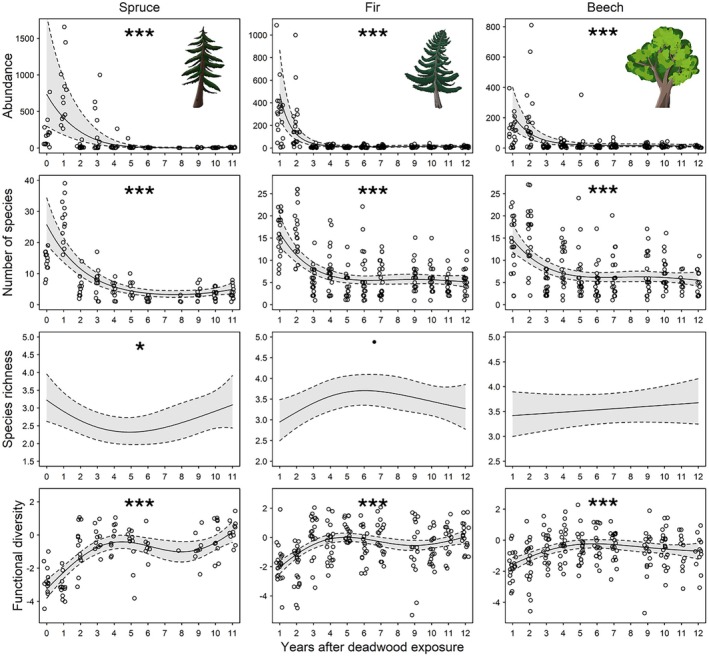
Predicted beetle abundance, number of species (species density; sensu Gotelli & Colwell, 2001), species richness (number of species controlled for abundance; sensu Gotelli & Colwell, 2001) and functional diversity (SES MFPD; Cadotte et al., 2013) as functions of years after deadwood exposure (numeric) across three tree species (spruce, fir and beech). Predictions are derived from generalized additive models (GAMs), with solid lines representing model‐predicted mean responses over years after deadwood exposure. Shaded bands depict 95% confidence intervals based on standard errors of predictions calculated on the link scale and back‐transformed to the response scale for abundance, number of species and species richness (modelled with a negative binomial distribution). For functional diversity (modelled with a Gaussian distribution and identity link), confidence intervals are based directly on the response scale without back‐transformation. Observed raw data points are overlaid, except for species richness (including log(abundance) as a fixed effect) where raw points of number of species are not shown due to scale mismatches. Statistical significance in the smooth term is indicated using asterisks and dots (*** = *p* < 0.001, * = *p* < 0.05 and · = *p* < 0.1).

The smoothed slopes of abundance and number of species declined over time for spruce, fir and beech. For fir and beech, these declines stabilized after ~4 years, while spruce showed a secondary increase after ~8 years of deadwood exposure. Species richness remained unchanged for beech, whereas spruce exhibited a U‐shaped pattern and fir a weak hump‐shaped response. Functional diversity increased for all tree species during the first 3 years and then plateaued. Around 10 years after exposure, an increase in functional diversity was observed for both conifers, more pronounced in spruce (Figures 1b and 2, Supporting Information Section 5: Table S3).

The trait‐exclusion analysis revealed that removing ‘host tree’ (i.e. conifer vs. broadleaf vs. both) (Δ*R* ^2^ = −0.060), ‘feeding type’ (i.e. xylophagous, mycetophagous and predatory) (Δ*R* ^2^ = −0.032) and ‘body length’ (Δ*R* ^2^ = −0.031) caused the largest reductions in explanatory power. These traits therefore had the strongest influence on functional diversity patterns. Exclusion of other traits resulted in minor changes (Δ*R* ^2^ ≤ 0.02), suggesting that their contribution was comparatively weak (Supporting Information Section 4: Table S2).

**FIGURE 3:**
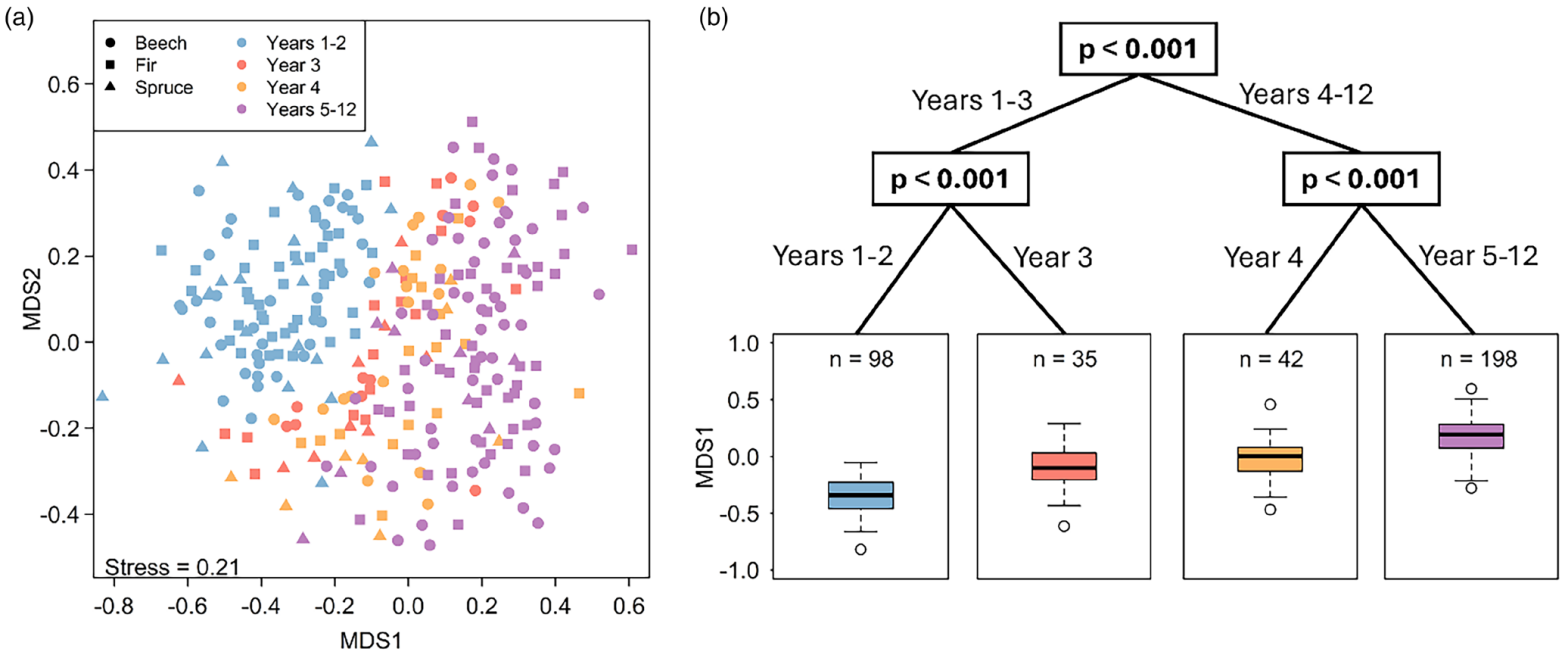
(a) NMDS ordination of saproxylic beetle community compositions emerging over 12 years from deadwood logs of spruce (triangles), fir (squares) and beech (circles). Each point represents the beetle assemblage emerging from a single deadwood log in a given year. Assemblages were included only if at least four beetle species emerged. Assemblages from the 0 year of spruce were excluded (see Section 2). Colours indicate distinct community compositions identified by year after deadwood exposure, as determined by the conditional inference tree. (b) Conditional inference tree illustrating significant differences in beetle community compositions across years. The conditional inference tree is based on the scores of the first NMDS axis (MDS1, see Section 2), and identifies distinct community compositions between years after deadwood exposure (treated as factor to capture year‐specific effects).

**FIGURE 4:**
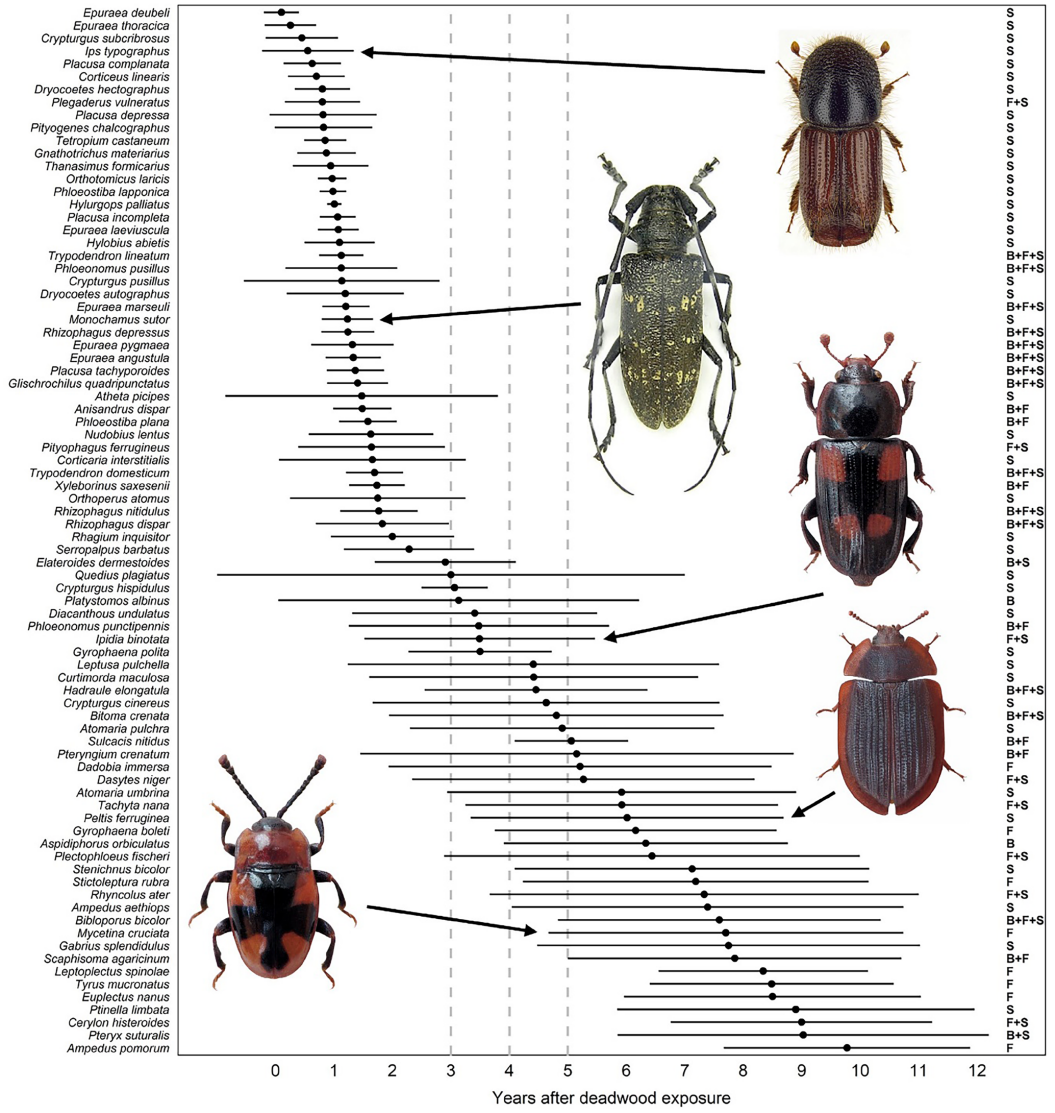
Temporal niche positions and breadths of beetle species in relation to deadwood exposure time. Points represent the abundance‐weighted mean year of emergence for each beetle species, calculated by averaging all emergence years weighted by the number of individuals per year. Horizontal lines represent standard deviation around the mean, reflecting the temporal spread of emergence (interpreted as niche breadth). Only species with at least three individuals emerging from any given deadwood log within a year were included for visualization. Tree species associations (i.e. from which tree species the beetle species emerged) are indicated by abbreviations on the right (B, beech; F, fir; S, spruce). The vertical grey dashed lines indicate the unique beetle compositions identified by the conditional inference tree for years 1–2, 3, 4 and 5–12. Photo credits: Jörg Müller.

The NMDS separated beetle assemblages over time (first axis) (Figure 3a). The conditional inference tree (based on the scores of the first axis) showed two distinct beetle assemblages overall between years 1 and 3, then after the 4th year. Community composition differed significantly across years, with years 1 and 2 forming a similar assemblage, years 3 and 4 each forming distinct assemblages and years 5–12 grouped together as one distinct assemblage (Figure 3b). Permanova revealed that both tree species (*R* ^2^ = 0.023, *p* < 0.001) and the year after deadwood exposure (*R* ^2^ = 0.143, *p* < 0.001), significantly influenced beetle community composition (Section 6 of Supporting Information Table S5). Beetle species showed a clear temporal niche pattern. Species with a narrow temporal niche appeared clustered for all tree species in the first years after deadwood exposure (Figure 4, Section 7 of Supporting Information Figure S6).

## 4 DISCUSSION

In our 12‐year experiment, we quantified community assembly patterns of saproxylic beetles in deadwood logs in temperate forests in light of the *ephemeral resource patch* (ERP) *concept*, revealing that patch‐scale characteristics of this intermediate‐longevity resource shape consumer communities. Across the studied tree species, beetle abundance, number of species and functional diversity followed similar trends, suggesting early communities were driven by the *more‐individuals hypothesis* and strong *habitat filtering*, comparable to patterns frequently observed in short‐lived ERPs. Beetle assemblages were more distinct in the early stage of decomposition, compared to advanced stages, indicating a strong early successional pattern in beetle assemblages. However, the prevalence of abundant narrow‐niched specialists was largely confined to spruce, which also showed stronger early functional filtering compared to beech and fir. There was limited support for the *habitat‐heterogeneity hypothesis* in terms of variation in beetle species richness among tree species.

Ephemerality is a defining characteristic of ERPs, which are displayed as distinct stages of decomposition in deadwood with intermediate longevity. While it is well established that decomposition is an important determinant of wood‐inhabiting species communities (Müller et al., 2020; Stokland et al., 2012), studies failed to track consumer communities on the same deadwood object through to near‐complete depletion (Seibold et al., 2023; Vindstad et al., 2020). Others lack continuous sampling or aggregate beetle assemblages over broad time intervals, limiting insight into fine‐scale community shifts (Jonsell et al., 2019; Neff et al., 2022). In line with our first hypothesis (H1), we observed high abundance and number of species of saproxylic beetles during the early decomposition stage, which is commonly observed in deadwood (Stokland et al., 2012; Ulyshen, 2018; Ulyshen & Hanula, 2010), likely due to energy‐rich phloem and cambium layers attracting early colonizers (Filipiak, 2018). After controlling the number of species for abundance, beetle species richness responses diverged among tree species: in beech, richness was fully explained by abundance (consistent with the *more‐individuals hypothesis*); in fir, beetle richness showed a weak early increase before levelling off (partial support for the *more‐individuals hypothesis*); and in spruce, beetle richness declined despite high abundance (no support for the *more‐individuals hypothesis*). Such high abundance and pronounced aggregation patterns have been described as typical for early ERP consumers (Benbow et al., 2019; Yang et al., 2008). The sharp early decline in both abundance and species number of saproxylic beetles across all three tree species supports the idea that fresh deadwood acts as a resource pulse (Yang et al., 2008). In our study, early colonizers included bark beetles (Curculionidae: Scolytinae), such as *Trypodendron lineatum*, *Xyleborinus saxesenii* and *Ips typographus*, which are known to colonize in large numbers (Lindgren & Raffa, 2013; Raffa et al., 2016; Sauvard, 2004).

Previous studies have demonstrated the consistent effects of finite resource heterogeneity on saproxylic beetle communities at both the landscape and patch scales (Lettenmaier et al., 2022; Müller et al., 2020; Seibold et al., 2016), as well as for short‐lived ERPs (Butterworth et al., 2022). However, our findings offer only weak support for the *habitat‐heterogeneity hypothesis* in advanced decomposition stages at the scale of a tree forming the local habitat patch (contradicting H2). Species richness remained stable in beech, followed a U‐shaped pattern in spruce and a weakly hump‐shaped pattern in fir. Butterworth et al. (2022) highlighted that ERPs vary in heterogeneity over time. In the early stages, these resources are often colonized by only a few highly specialized species capable of overcoming physical barriers (e.g. bark) and chemical defences. While species richness is often used as a proxy for habitat heterogeneity (i.e. the number of available niches), it may be misleading when generalist species dominate (Butterworth et al., 2022). This pattern is reflected in our analysis of beetle species' temporal niche positions and breadths, where early colonizers tend to have narrower temporal niches, while later‐arriving species exhibit broader temporal niches across advanced decomposition stages. While factors such as increased fungal resources for beetles (Rajala et al., 2012, 2015), structurally diverse woody debris (Přívětivý & Šamonil, 2021) and higher temperature heterogeneity (Lettenmaier et al., 2022) may increase habitat heterogeneity, they did not appear to have a strong enough influence to significantly increase beetle species richness in our study. Seibold et al. (2016) found that positive relationships between beetle richness and both the amount and diversity of deadwood were consistent with the *habitat‐heterogeneity hypothesis* when examining some of the same deadwood logs at forest stand scale (i.e. beetles sampled by flight interception traps). This suggests that greater habitat variety at a larger scale (i.e. forest stand or landscape scale) can create more niches, leading to higher beetle richness as opposed to local heterogeneity found on a single log. Our study did not investigate landscape‐scale characteristics and therefore cannot directly evaluate the observation of Seibold et al. (2016). However, at the level of individual deadwood logs, factors like fungal diversity and abiotic heterogeneity between deadwood logs seem to influence beetle species richness only at certain stages of decomposition and not in the same way across different tree species. The traps cover an area of ≈0.35 m^2^, and it has been reported that emerging arthropods are sampled as accurately as in situ rearing from experimental logs (Hagge, Leibl, et al., 2019). However, Hagge, Leibl, et al. (2019) sampled deadwood only in the first year after exposure, which is not comparable to the heterogeneous wood in advanced stages. Therefore, the limited area of 0.35 m^2^ may not be sufficient to represent the heterogeneity of the stem in advanced stages of decomposition. Another potential explanation for the inconsistent species richness patterns in our study may be the result of stochastic processes in intermediate‐longevity resources (Pulsford et al., 2016; Seibold et al., 2023), which do not apply to very short‐lived ERPs. For instance, early colonizers and their mutualists can shape the occurrence of later saproxylic species or communities (Dickie et al., 2012; Fukami et al., 2010; Jacobsen et al., 2015; Weslien et al., 2011) by either modifying the physical structures and food resources, or the amount of niche resources (niche preemption), therefore changing niche availability to later‐successional species (priority effect) (Fukami, 2015). One illustration of this is the resource redistribution by fungal mutualists and antagonists in different parts of the wood (Six & Elser, 2019), which could influence habitat heterogeneity within the wood and ultimately the species richness of beetles. Furthermore, interactions among beetles at the same time can lead to altered colonization dynamics through competition and facilitation (Joensuu et al., 2008; Victorsson, 2012; Zuo, Cornelissen, et al., 2016). For example, entry holes created by beetles facilitate other saproxylic beetles to enter the deadwood (Buse et al., 2008; Zuo, Cornelissen, et al., 2016).

Our results support our hypotheses H3 and H4, which predicted a low functional diversity in the early decomposition stage, indicating strong *habitat filtering* and an increase in functional diversity in advanced stages (weak *habitat filtering*). Saproxylic beetles face multiple filters that exclude species with specific traits unsuited for deadwood colonization, operating across spatial scales: (1) from a region to site (plot); (2) from site to patch (subplot where deadwood logs were placed) and (3) finally colonizing the deadwood resource (Neff et al., 2022). Neff et al. (2022) showed that each of these steps along spatial scales (i.e. region to site to patch and finally to object scale) resulted in a loss of functional diversity of saproxylic beetles and shifted the composition of beetle assemblages. From the site to patch step, saproxylic beetles have to be able to detect and evaluate potential host trees prior to arrival (Graf et al., 2022), highlighting once more the importance of landscape characteristics of ERPs (Butterworth et al., 2022). By the last step, that is, colonizing the fresh deadwood log, the filtering effects add up, resulting in beetle assemblages that only include functionally similar species. Resource tracking, adaptation in resource detection and dispersal are key evolutionary processes that have shaped the diversity of colonizers across short‐lived ERPs (Butterworth et al., 2022). Additionally, host‐tree preference was the strongest driver for the functional diversity of our studied beetle assemblages, highlighting the deep evolutionary link between beetles and their host trees (Burner et al., 2021; Müller et al., 2020). This link is evident in the beetles' successful morphological adaptations, which allow them to exploit the high‐energy nutrients present in the early stages of deadwood decomposition. Breaking through tough physical barriers like bark (Hagge, Bässler, et al., 2019; Zuo, Berg, et al., 2016) and hardwood therefore is an important filtering role in morphological adaptations for saproxylic beetles (Hulcr et al., 2015; Six, 2012). Additionally, feeding type (i.e. xylophagous, mycetophagous and predatory) was the second most important driver for functional diversity, highlighting the role of deadwood in shaping trophic diversification. This, in turn, contributes to the evolutionary diversification of consumers, a principle commonly observed in short‐lived ERPs (Butterworth et al., 2022). Besides filtering for deadwood colonization, the presence of high amounts of chemical compounds in the early stages also plays a role. This causes beetles to adapt, such as mutualism with microbes, thus attracting more specialized species (Biedermann & Vega, 2020; Six, 2012; Stokland et al., 2012; Wende et al., 2017). ERP consumers often form symbiotic relationships with microbes, which can increase resource consumption or improve its longevity and quality (Butterworth et al., 2022). Many of the beetle species in our study are ambrosia beetles, which are known to introduce fungi that alter the composition of deadwood, benefiting themselves and other invertebrates while prolonging the decay process (Skelton et al., 2019).

Our results provide strong evidence that saproxylic beetle communities in deadwood follow a structured successional trajectory shaped by the *more‐individuals hypothesis* and *habitat filtering*, with only limited support for the *habitat‐heterogeneity hypothesis*. The significant influence of time since deadwood exposure on community composition led to the formation of two major assemblage groups: an early phase (years 1–3) and a later phase (years 4–12). Within this trajectory, years 1 and 2 formed a similar assemblage, years 3 and 4 were compositionally distinct and years 5–12 grouped into a stable, advanced‐decomposition assemblage. Temporal niche patterns further support this successional framework. Early colonizing beetle species showed narrow temporal niche breadths, suggesting specialization on the early decay stages, while later‐stage species displayed broader emergence periods. These findings support H5, highlighting distinct community compositions in the early decomposition stage compared to advanced stages. Importantly, the strong community shifts in early successional dynamics reflect the succession principle frequently observed in short‐lived ERPs (Butterworth et al., 2022), such as carrion (von Hoermann et al., 2023). We argue that deadwood, despite its intermediate longevity, exhibits early‐stage dynamics that closely resemble those of short‐lived ERPs, driven by a sharp pulse of available energy and strong filtering pressures. However, the extended decomposition period of deadwood logs allows for a transition to a more stable consumer community, extending beyond the classical ERP framework. Collecting insects from deadwood is challenging, as wood has an irregular surface and increasingly develops cracks as it decomposes, making perfect trap sealing difficult (Wende et al., 2017). Alternative methods, such as extraction tubes, alter microclimate and prevent recolonization (Hagge, Leibl, et al., 2019). Although, we carefully sealed and regularly inspected traps throughout the study, we cannot fully exclude the possibility that sealing efficiency declined in later decomposition stages. Such effects would primarily affect the final years, when mechanical decomposition was most advanced. However, a critical evaluation of the beetle species collected showed no evidence of contamination of species composition over time or between tree species (Möller, 2009). We are therefore confident that our assemblages reflect beetle emergence reliably across the 12 years, and that any sampling limitations are confined to the late stages without affecting the robustness of our earlier findings.

## 5 CONCLUSION

Building on previous research and insights from our 12‐year continuous and replicated decomposition experiment with three tree species, we demonstrate that deadwood follows key principles of the ephemeral resource patch (ERP) concept. By testing the *more‐individuals hypothesis*, the *habitat‐heterogeneity hypothesis* and *habitat filtering* theory, we show that the early stages of deadwood decomposition closely resemble those of short‐lived ERPs, such as carrion, with rapid community turnover driven by changes in habitat conditions and resource quality. These early successional dynamics were largely consistent across tree species, although beetle species richness showed tree‐specific differences, highlighting the overarching role of temporal continuity in deadwood availability. Forest management strategies should therefore consider not only the quantity but also the temporal continuity of deadwood input to support the full spectrum of saproxylic beetle communities. This does not imply increasing logging activity but rather ensuring that when deadwood is already being created, whether through natural mortality or existing forest management, it occurs in a staggered manner over time. Moreover, our findings refine the conceptual framework of ERPs by illustrating that intermediate‐longevity resources, such as deadwood, transition from an early, resource‐pulse‐driven stage to a more stable phase, during which they no longer function as ERPs sensu stricto, even though they are not yet fully depleted. In this intermediate stage, deadwood continues to provide important habitat and structural complexity, despite its diminishing role as a temporally finite resource. This dual character highlights that intermediate‐longevity resources operate both within the ERP framework during early decomposition and beyond it in later stages, functioning as longer‐term habitat.

## AUTHOR CONTRIBUTIONS

Jörg Müller and Ludwig Lettenmaier conceived the ideas and designed methodology; Ludwig Lettenmaier, Orsi Decker, Jonas Hagge, Christoph Heibl, Giorgi Mamadashvili, Sebastian Seibold and Simon Thorn collected the data; Ludwig Lettenmaier analysed the data; Ludwig Lettenmaier and Jörg Müller led the writing of the manuscript. All authors contributed critically to the drafts and gave final approval for publication.

## CONFLICT OF INTEREST STATEMENT

The authors declare no conflicts of interest.

## Supporting information


**Section 1.** Experimental setup.
**Section 2**. Traits.
**Section 3**. Phylogeny.
**Section 4**. Calculation of mean functional‐phylogenetic distance (MFPD) and trait exclusion analysis.
**Section 5**. GAM models.
**Section 6**. Permanova.
**Section 7**. Temporal niche breadth position.
**Section 8**. Venn diagram.

## Data Availability

Data available from Zenodo https://doi.org/10.5281/zenodo.17337484 (Lettenmaier et al., 2025).
