# Supplementary material for: 12 years of assembly patterns in saproxylic beetles suggest early decay wood as ephemeral resource patch
Source: J Anim Ecol. 2025 Nov 11;95(2):282–95. doi: 10.1111/1365-2656.70183 (PMC12868409; doi:10.1111/1365-2656.70183)
Supplement: Supplementary file 1 — Section 1. Experimental setup. Section 2. Traits. Section 3. Phylogeny. Section 4. Calculation of mean functional‐phylogenetic distance (MFPD) and trait exclusion analysis. Section 5. GAM models. Section 6. Permanova. Section 7. Temporal niche breadth position. Section 8. Venn diagram. [file JANE-95-282-s001.docx]

**Supporting Information for:**

**12 years of assembly patterns in saproxylic beetles suggest early decay wood as ephemeral resource patch**

Ludwig Lettenmaier^1,2,*^, Claus Bässler^2,3^, Orsi Decker^2^, Jonas Hagge^4,5^, Christoph Heibl^2^, Giorgi Mamadashvili^1^, Sebastian Seibold^6^, Simon Thorn^7,8,9^, Jörg Müller^1,2^

**^1^**Field Station Fabrikschleichach, Chair of Conservation Biology and Forest Ecology, Biocenter, University of Würzburg, Rauhenebrach, Germany

^2^Bavarian Forest National Park, Freyunger Str. 2, 94481 Grafenau, Germany

^3^University of Bayreuth, Fungal Ecology, Bayreuth Center of Ecology and Environmental Research (BayCEER), Universitätsstrasse 30, 95440 Bayreuth

^4^Northwest German Forest Research Institute, Department of Forest Nature Conservation, Hann. Münden, Germany

^5^University of Göttingen, Department of Forest Nature Conservation, Göttingen, Germany

^6^TUD Dresden University of Technology, Forest Zoology, Pienner Str. 7, 01737 Tharandt

^7^Hessian Agency for Nature Conservation, Environment and Geology, State Institute for the protection of birds, Netanyastraße 5, 35394 Gießen, Germany

^8^Philipps Universität Marburg, Applied Ecology, Karl-von-Frisch-Straße 8, D-35043 Marburg

^9^Czech Academy of Sciences, Biology Centre, Institute of Entomology, Branišovská 1160/31, CZ-37005 České Budějovice, Czech Republic

*Corresponding author: Ludwig Lettenmaier [lettenmaierludwig@gmail.com](mailto:ludwig.lettenmaier@uni-wuerzburg.de)

**Table of Contents:**

| **Section 1: Experimental setup** | Page 2 |
| --- | --- |
| **Section 2: Traits** | Page 4 |
| **Section 3: Phylogeny** | Page 7 |
| **Section 4: Calculation of mean functional-phylogenetic distance**  **(MFPD) and trait exclusion analysis** | Page 9 |
| **Section 5: GAM models** | Page 11 |
| **Section 6: Permanova** | Page 13 |
| **Section 7: Temporal niche breadth position** | Page 14 |
| **Section 8: Venn diagram** | Page 15 |
| **References** | Page 16 |

# **Section 1: Experimental setup**


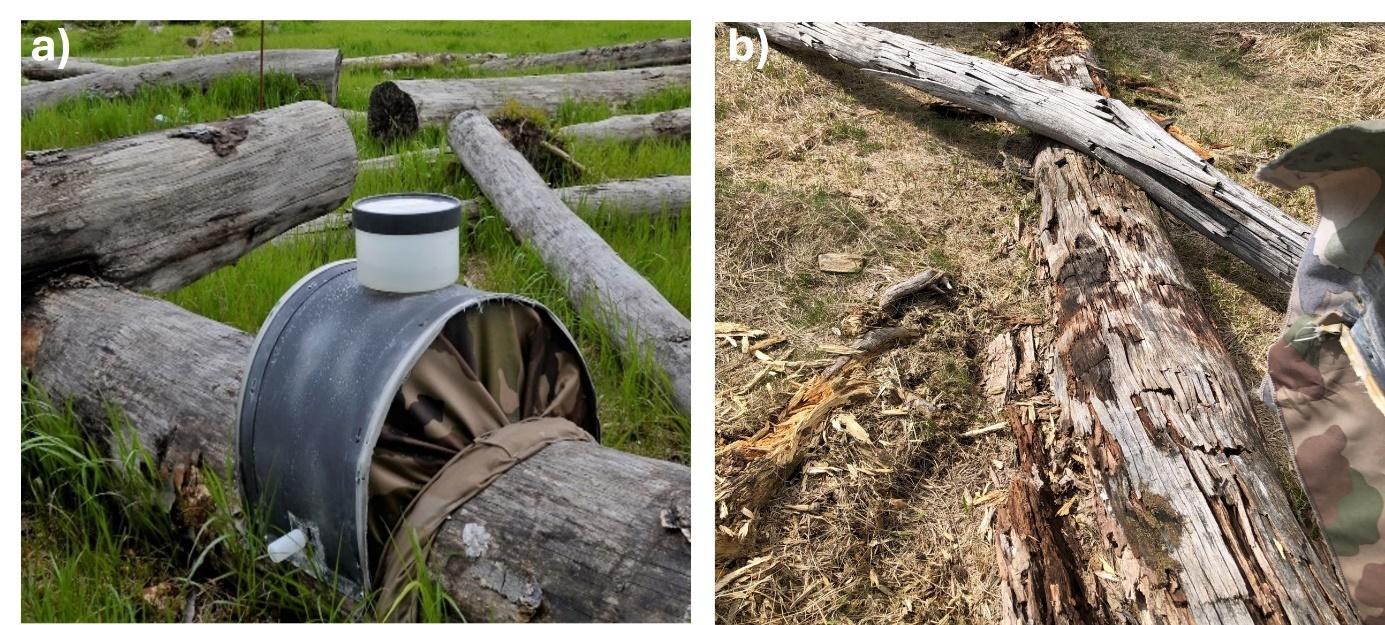


**Figure S1**: **a)** A stem emergence trap placed on an experimental deadwood log (5 years after exposure) to capture beetles emerging from the wood. **b)** An example of one of our experimental deadwood logs (12 years after exposure) that can no longer support the attachment of a stem emergence trap, marking the end of the study period. Photo credits: Ludwig Lettenmaier.

We used data from saproxylic beetles sampled from deadwood logs from two different deadwood experiments (Seibold et al., 2016; Thorn et al., 2016). Both experiments were conducted in the montane zone of the Bavarian Forest National Park, located in southeastern Germany. The forested area consists of both broadleaf and coniferous trees, creating a mosaic of closed-canopy forests interspersed with sunny gaps caused by bark beetle activity (Müller et al., 2010). The first experiment was initiated in autumn 2011, when 190 plots, each measuring 0.1 ha, were randomly established within five blocks across the study area (Seibold et al., 2016). Half of the plots were placed in sunny clearings, while the other half were situated under a closed forest canopy. To avoid die-back of the canopy due to bark beetle infestations, closed canopy plots were positioned under European beech (*Fagus sylvatica*). The surrounding forest areas for both sunny and shady plots were composed of a mix of broadleaf and coniferous species. Plots were randomly assigned one of 19 different treatments designed to create variations in both the amount and diversity of deadwood. The wood used was cut within 8 weeks of placement and included logs (~ 30 cm in diameter, 5 m in length) from European beech and/or silver fir (*Abies alba*) and/or branches (3–5 cm in diameter, 2–3 m in length) from one or both tree species. Along with a control plot where no wood was added, each treatment included either a low or high amount of branches (8 branches, about 0.2 m³ ha⁻¹, or 80 branches, about 2 m³ ha⁻¹) or logs (4 logs, about 10 m³ ha⁻¹, or 40 logs, about 100 m³ ha⁻¹), or combinations of logs and branches at either low or high amounts. The deadwood diversity gradient was formed by combining different wood types at three levels of diversity. The lowest diversity level involved a single example of each substrate type (beech logs, beech branches, fir logs, fir branches), the intermediate level combined different diameter classes from the same tree species (beech logs and branches, fir logs and branches), or used one diameter class of both species (beech and fir logs, beech and fir branches). The highest diversity level used both tree species with logs and branches. Half of the logs were placed on top of one another, with some in full contact with the soil and others elevated, making them drier. Only logs with full ground contact had stem emergence traps attached to them. We selected plots that contained only deadwood logs with either logs of beech or fir or both. The final data set comprised 20 beech and 20 fir logs. The second experiment started in April 2013 (Thorn et al., 2016). Twelve artificial windthrow sites in the northern region of the Bavarian Forest National Park were created. Six plots were placed in densely forested areas with high canopy cover. The remaining six plots were placed in sun-exposed areas, resulting in plot pairs with a minimum distance of 200 meters between each open and closed plot. As in the first experiment, the forest had the same structural and compositional characteristics. In April 2013, three mature spruce trees (*Picea abies*) on each plot were mechanically uprooted using steel cables and winches to simulate natural storm events. One tree per plot was left intact as a control, while the other two trees were severed from their root plates, with branches cut and left on the ground to facilitate subsequent mechanical bark treatments. We only used the beetles sampled with stem-emergence traps from the control logs (not treated). Therefore, each plot contained one spruce log, resulting in 12 spruce logs.

# **Section 2: Traits**

Flightless species (n = 5) missed wing traits and therefore were replaced by zero which corresponded to the null expectation of residuals. For 27 species, one or more morphological traits were missing. We estimated missing values by averaging the respective trait value from all species within the same genus that were sampled in our study (Neff et al., 2022). In cases where no other species from the same genus were part of our study, or if the particular trait was also absent in those species, we determined the mean based on the trait information provided by Hagge et al. (2021). For 16 species, one or more ecological traits were missing. For numerical ecological traits (decay niche and wood diameter niche) we used the same estimation procedures as for morphological traits. However, for categorical ecological traits (feeding type and host trees) we used a twofold approach. First, we visualized the frequency of occurrence of each species in different years after deadwood exposure and their frequency in different tree species. Second, we examined the respective ecological traits of sister species. After evaluating this combined information, an expert provided validation. Low number of species with distinct categorical traits can lead to an unbalanced representation, and therefore, potential bias our analyses. Therefore, we reclassified *Attagenus schaefferi* and *Megatoma undata* from detritivore to xylophagous.

**Table S1:** Summary of the 13 morphological and 4 ecological traits used in the analysis. Due to high correlations among certain morphological traits and body size, several traits were either standardized by body length or left unstandardized (for more details see Hagge et al. 2021). Color lightness was calculated as the average of RGB values, scaled from 0 (pure black) to 255 (pure white). All trait values shown are standardized.

|  | | |  | **Transformation** | |  |
| --- | --- | --- | --- | --- | --- | --- |
| **Trait** | | | **Range** | **Body length** | **Logarithm** | **Reference** |
| **Morphological traits** | **Body** **shape** | Body length | 0.592 – 3.086 |  | x | Hagge et al. (2021) |
|  |  | Body width | -1.028 – 0.565 | x |  | Hagge et al. (2021) |
|  |  | Body roundness | 0.385 – 1.118 |  |  | Hagge et al. (2021) |
|  |  | Head length | -0.747 – 1.045 | x |  | Hagge et al. (2021) |
|  | **Locomotion** | Wing length | -0.792 – 0.545 | x |  | Hagge et al. (2021) |
|  |  | Wing aspect | 0 – 3.747 |  |  | Hagge et al. (2021) |
|  |  | Wing load | -2.166 – 1.432 | x |  | Hagge et al. (2021) |
|  |  | Leg length (front femur) | -0.773 – 0.791 | x |  | Hagge et al. (2021) |
|  | **Sensory** | Antenna length | -1.090 – 2.277 | x |  | Hagge et al. (2021) |
|  |  | Eye length | -0.727 – 1.077 | x |  | Hagge et al. (2021) |
|  |  | Hairiness | 0 – 4.927 |  | x | Hagge et al. (2021) |
|  | **Foraging** | Mandibular aspect | 0.103 – 1.292 |  |  | Hagge et al. (2021) |
|  | **Colour** | Lightness | 81.840 – 148.50 |  |  | Hagge et al. (2021) |
| **Ecological traits** | | Decay niche | 1 – 4.5 (alive – decomposed) |  |  | Gossner et al. (2013) |
|  |  | Wood diameter niche | 1 – 4 (small – large) |  |  | Gossner et al. (2013) |
|  |  | Feeding type | xylophagous, mycetophagous, predatory |  |  | Köhler (2015),  Seibold et al. (2015) |
|  |  | Host tree | conifer, broad-leaved, both |  |  | Köhler (2015),  Seibold et al. (2015) |


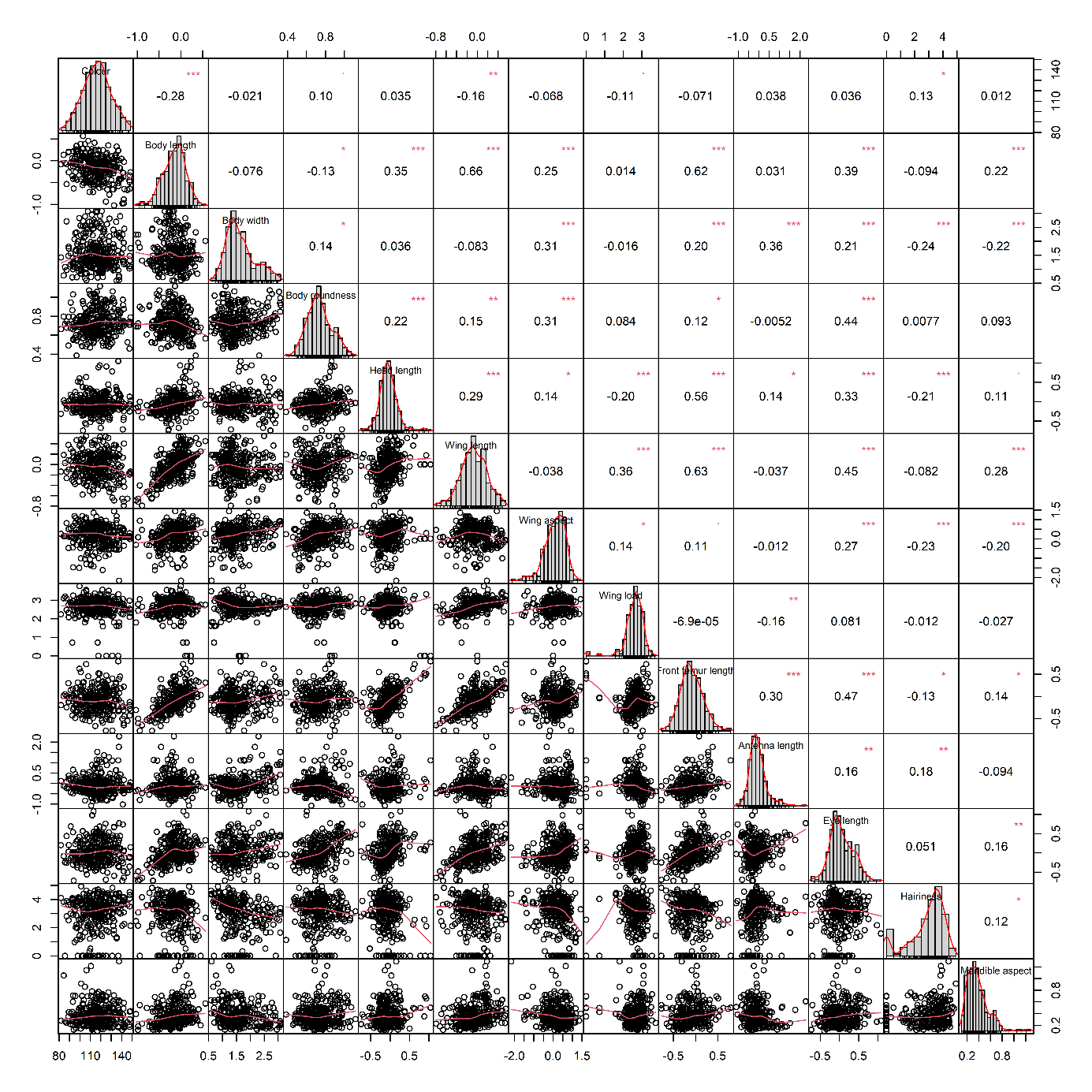


**Figure S2**: Pearson’s correlation of the 13 morphological traits (above the diagonal; p < 0.05 = *, p < 0.01 = **, p < 0.001 = ***)

# **Section 3: Phylogeny**

Three missing species were inserted as sister species that did not appear in the sampled species in this study (i.e. they were replaced). *Corticaria pineti was* inserted next to *C. lapponica*, *Orthoperus rogeri* next to *O. brunnipes* and *Phloeopora scribae* next to *P. teres*). *Placusa complanate* could be added into the phylogeny at the base of genus *Placusa*. *Micridium halidaii* is a member of Ptilidae and had no congenerics in the phylogeny. Polilov et al. (2019) present the first comprehensive phylogenetic analyses of morphological and molecular data and on page 450 they state: Ptiliinae are not confirmed as a clade. Ptiliola and Ptiliolum are placed in a polytomy with other ptiliid lineages in the morphological trees. However, Actidium, Oligella and Micridium + Ptilium form a reasonably well supported monophylum when using morphological data, characterized by a specific shape of the mentum, a pronotum widest in the anterior region, and a shortened and moderately flattened penis”. Therefore, we chose to add *Micridium halidaii* as a sister of *Ptiliolum caledonicum* to the phylogeny. Comparing to the results in Polilov et al. (2019) it became clear that the topology representing Ptiliidae in the phylogeny is probably not correct. At the same time, their topologies are not sufficiently resolved to propose a correction.


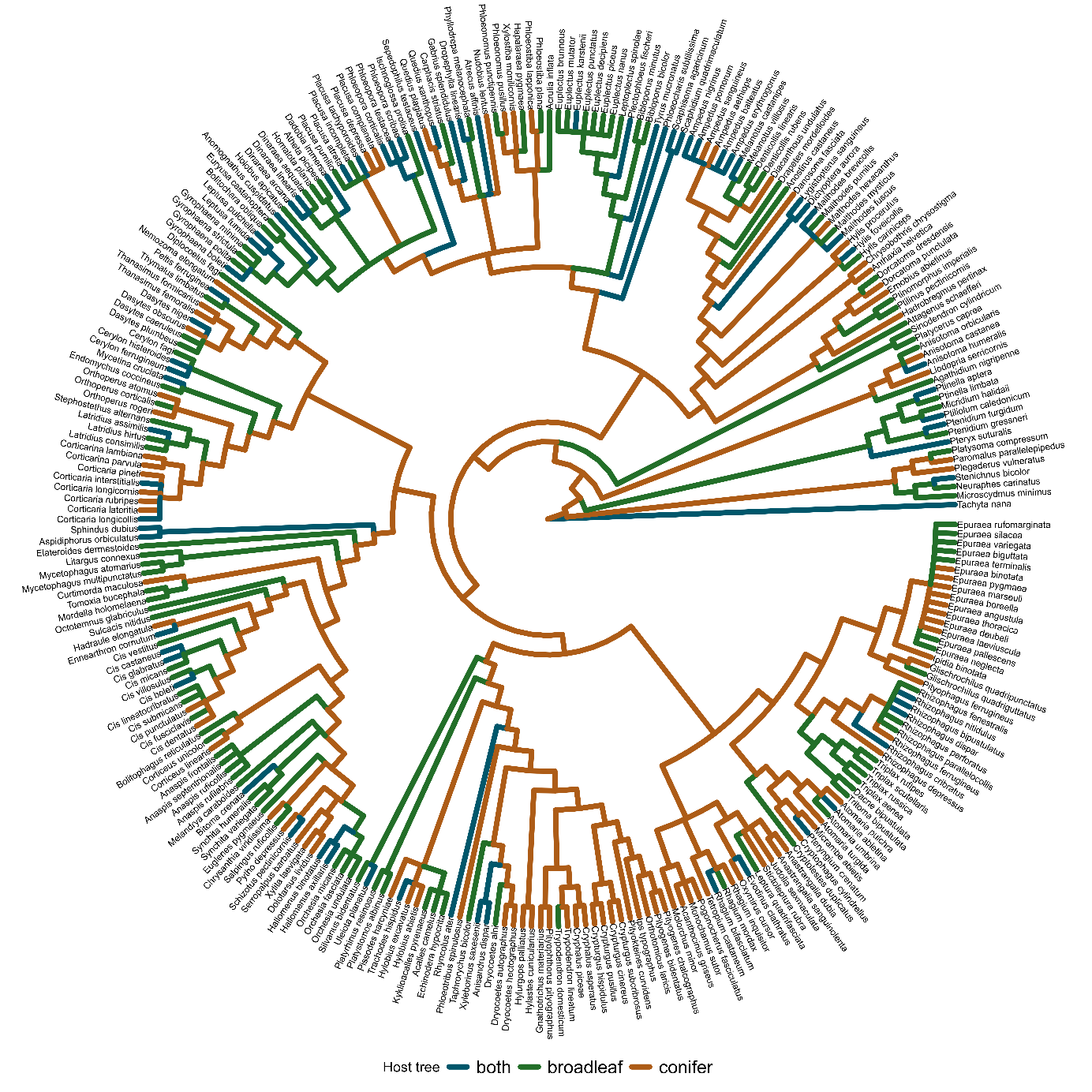


**Figure S3:** Cladogram of the 297 saproxylic beetle species. Branch colors correspond to different host tree preferences. The tree topology is based on Hunt et al. (2007) and adapted by Hagge et al. (2021). For this study, five species were additionally added (*Corticaria pineti*, *Micridium halidaii*, *Orthoperus rogeri*, *Phonopores scribae* and *Placusa complanate*). The illustration was created with the *ggtree* package (Yu et al., 2017).

# **Section 4: Calculation of mean functional-phylogenetic distance (MFPD) and trait exclusion analysis**

We assessed mean functional-phylogenetic distance (MFPD) following the framework of Cadotte et al. (2013), which integrates functional and phylogenetic distances through a weighting parameter *a*. This parameter determines the relative contribution of each component: when *a* = 0, only functional distance is considered; when *a* = 1, only phylogenetic distance is included. Intermediate values reflect a combined influence of both. Functional distances among species were calculated using Gower distance, which accommodates both categorical and continuous data types, via the daisy function from the cluster package (Maechler et al., 2022). Phylogenetic distances were derived from a species-level tree using the cophenetic function in the stats (R Core Team, 2023).

To control for variation in species richness across samples and to isolate signals of habitat filtering, we applied a null model approach using tip-shuffling (Cadotte & Davies, 2016). This produced standardized effect sizes (SES) for the mean pairwise functional-phylogenetic distance (hereafter referred to as “functional diversity”) calculated from 999 randomizations with the ses.mpd function in the picante package (Kembel et al., 2010). We modelled functional diversity (SES MFPD) using generalized additive models with a Gaussian error distribution and identity link function. The model included a smooth term for years since deadwood exposure, fitted separately for each tree species (s(years after deadwood exposure, by = tree species, k = 4)), a fixed effect for tree species, and a spatial smooth term for coordinates. To determine the most informative combination of functional and phylogenetic distances, we ran models across a range of *a*-values (0 to 1, in 0.025 intervals). The optimal value (*a* = 0.35) was selected based on the highest adjusted R² (Fig. S4).


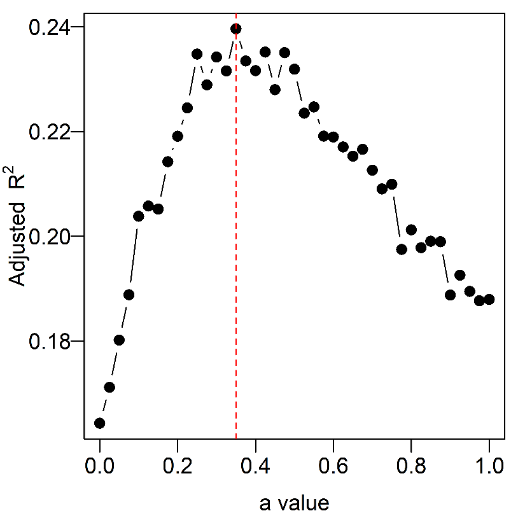


**Figure S4:** Adjusted R² values from 41 generalized additive models fitted across a gradient of a -values ranging from 0 to 1 in 0.025 intervals. The a-parameter controls the relative weighting of functional versus phylogenetic distances in the mean functional-phylogenetic distance. Higher adjusted R² values indicate better model fit.

To assess the contribution of individual traits to functional diversity, we performed a trait-exclusion analysis. For each iteration, we recalculated mean functional–phylogenetic distance (MFPD) with α = 0.35 while omitting one trait at a time, and refitted the generalized additive model. We then compared the adjusted R² values of the reduced models to that of the full model (R² = 0.243). A larger reduction in adjusted R² (ΔR²) indicated a stronger contribution of the excluded trait to shaping community functional structure (Table S2).

**Table S2:** Adjusted R² values from generalized additive models with functional diversity (SES MFPD) as the response variable. Distance matrices were recalculated by iteratively excluding one trait at a time using an α-value of 0.35. The table reports adjusted R² for each reduced model and the change relative to the full model (R² = 0.243).

| **Excluded Trait** | **Adjusted R²** | **ΔR² relative to full model** |
| --- | --- | --- |
| Host tree | 0.183 | –0.060 |
| Feeding type | 0.211 | –0.032 |
| Body length | 0.212 | –0.031 |
| Niche decay | 0.219 | –0.024 |
| Niche diameter | 0.232 | –0.011 |
| Lightness | 0.222 | –0.021 |
| Antenna length | 0.238 | –0.005 |
| Head length | 0.240 | –0.003 |
| Mandible aspect | 0.241 | –0.002 |
| Body width | 0.241 | –0.002 |
| Wing aspect | 0.245 | +0.002 |
| Wing load | 0.246 | +0.003 |
| Hairiness | 0.254 | +0.011 |
| Eye length | 0.268 | +0.025 |
| Body roundness | 0.271 | +0.028 |
| Leg length | 0.275 | +0.032 |
| Wing length | 0.284 | +0.041 |

# **Section 5: GAM models**

*Abundance model*:

gam(number of individuals ~ s(years after deadwood exposure, by = tree species, k = 4) + tree species + s(E ,N, bs = "tp"), family=nb(theta = NULL, link = "log"), method= "REML", data = data))

*Species number model*:

gam(number of species ~ s(years after deadwood exposure, by = tree species, k = 4) + tree species + s(E ,N, bs = "tp"), family=nb(theta = NULL, link = "log"), method= "REML", data = data))

*Species richness model*:

gam(number of species ~ log(abundance) + s(years after deadwood exposure, by = tree species, k = 4) + tree species + s(E ,N, bs = "tp"), family=nb(theta = NULL, link = "log"), method= "REML", data = data))

*Functional diversity model*:

gam(SES MFPD ~ s(years after deadwood exposure, by = tree species, k = 4) + tree species + s(E ,N, bs = "tp"), family=gaussian(link = “identity”, data = data))

**Table S3:** Statistical results obtained from the gam models. All models included years after deadwood exposure (as numeric value) and factor-smooth interaction with tree species as a smooth function and coordinates of the logs as an additional smooth function to account for the repeated measurements. Tree species were additionally fitted as fixed effect. The logarithm of abundance was fitted as fixed effect for the species richness model. Functional diversity model included SES MPD extracted from null model approach as response variable. Significant values are indicated in bold and by asterisks (** = p < 0.05, *** = p < 0.001).

| **Model** | **Component** | **Term** | **Estimate** | **Std Error** | **z-value** | **p-value** |
| --- | --- | --- | --- | --- | --- | --- |
| **Abundance** | Parametric coefficients | (Intercept) | 3.202 | 0.107 | **29.998** | **< 0.001***** |
|  |  | Fir | -0.088 | 0.105 | -0.841 | 0.400 |
|  |  | Spruce | -0.094 | 0.373 | -0.253 | 0.800 |
|  | **Component** | **Term** | **edf** | **Ref. df** | **Chi. sq** | **p-value** |
|  | Smooth terms | s(years):Beech | 2.818 | 2.974 | **156.058** | **< 0.001***** |
|  |  | s(years):Fir | 2.946 | 2.998 | **292.394** | **< 0.001***** |
|  |  | s(years):Spruce | 2.906 | 2.992 | **348.734** | **< 0.001***** |
|  |  | s(E, N) | 16.907 | 20.746 | **76.708** | **< 0.001***** |
| **Model** | **Component** | **Term** | **Estimate** | **Std Error** | **z-value** | **p-value** |
| **Species number** | Parametric coefficients | (Intercept) | 2.021 | 0.044 | **45.460** | **< 0.001***** |
|  |  | Fir | -0.048 | 0.059 | -0.823 | 0.411 |
|  |  | Spruce | -0.229 | 0.102 | **-2.239** | **0.025**** |
|  | **Component** | **Term** | **edf** | **Ref. df** | **Chi. sq** | **p-value** |
|  | Smooth terms | s(years):Beech | 2.712 | 2.936 | **54.554** | **< 0.001***** |
|  |  | s(years):Fir | 2.790 | 2.965 | **84.902** | **< 0.001***** |
|  |  | s(years):Spruce | 2.669 | 2.911 | **149.672** | **< 0.001***** |
|  |  | s(E, N) | 6.933 | 8.849 | **25.348** | **0.003** |
| **Model** | **Component** | **Term** | **Estimate** | **Std Error** | **z-value** | **p-value** |
| **Species richness** | Parametric coefficients | (Intercept) | 25.348 | 0.053 | **17.777** | **< 0.001***** |
|  |  | log(abundance) | 0.385 | 0.014 | **26.657** | **< 0.001***** |
|  |  | Fir | -0.035 | 0.041 | -0.864 | 0.388 |
|  |  | Spruce | -0.286 | 0.063 | **-4.524** | **> 0.001***** |
|  | **Component** | **Term** | **edf** | **Ref. df** | **Chi. sq** | **p-value** |
|  | Smooth terms | s(years):Beech | 1.000 | 1.000 | 0.550 | 0.458 |
|  |  | s(years):Fir | 2.220 | 2.594 | 7.124 | 0.066 |
|  |  | s(years):Spruce | 2.201 | 2.534 | **9.683** | **0.023***** |
|  |  | s(E, N) | 2.001 | 2.002 | **6.105** | **0.047***** |
| **Model** | **Component** | **Term** | **Estimate** | **Std Error** | **z-value** | **p-value** |
| **Functional diversity** | Parametric coefficients | (Intercept) | -0.697 | 0.087 | **-7.971** | **< 0.001** |
|  |  | Fir | 0.081 | 0.121 | 0.671 | 0.503 |
|  |  | Spruce | -0.109 | 0.169 | -0.644 | 0.520 |
|  | **Component** | **Term** | **edf** | **Ref. df** | **Chi. sq** | **p-value** |
|  | Smooth terms | s(years):Beech | 2.613 | 2.888 | **8.729** | **< 0.001***** |
|  |  | s(years):Fir | 2.897 | 2.991 | **18.341** | **< 0.001***** |
|  |  | s(years):Spruce | 2.857 | 2.983 | **25.384** | **< 0.001***** |
|  |  | s(E, N) | 2.001 | 2.002 | 2.855 | 0.059 |

We tested for spatial autocorrelation in residuals of all GAM models (abundance, species number, species richness, and functional diversity) using Moran’s *I* tests implemented in the testSpatialAutocorrelation() function of the *DHARMa* R package (Hartig, 2022). The test calculates Moran’s *I* on distance-based spatial weights derived from plot coordinates to assess spatial structure in model residuals. None of the models showed significant spatial autocorrelation (Table S3).

**Table S4:** Results of Moran’s I tests for spatial autocorrelation of model residuals.

| **Response variable** | **Moran’s I** | **Expected** | **SD** | **p-value** |
| --- | --- | --- | --- | --- |
| **Abundance** | 0.058 | -0.020 | 0.072 | 0.281 |
| **Species number** | -0.061 | -0.020 | 0.073 | 0.572 |
| **Species richness** | -0.093 | -0.020 | 0.073 | 0.315 |
| **Functional diversity** | -0.079 | -0.021 | 0.071 | 0.414 |

# **Section 6: Permanova**

**Table S5:** Permanova results for the effects of tree species and year after deadwood exposure on saproxylic beetle community composition. Significant values are indicated in bold and by asterisks (*** = p < 0.001).

|  | **Df** | **SumOfSqs** | **Partial R^2^** | **F** | **p-value** |
| --- | --- | --- | --- | --- | --- |
| **Tree species** | 2 | 4.257 | 0.026 | 5.333 | **< 0.001***** |
| **Year after deadwood exposure** | 10 | 23.847 | 0.143 | 6.200 | **< 0.001***** |

# **Section 7: Temporal niche breadth position**


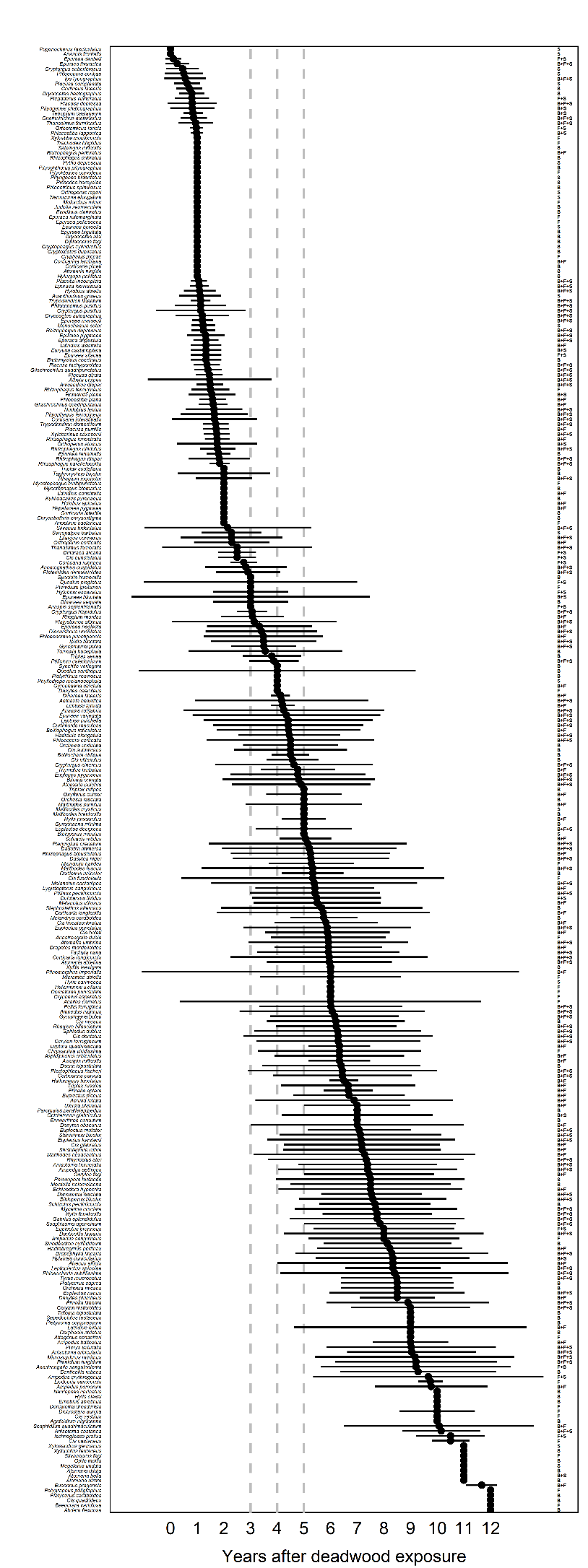


**Figure S6**: Temporal niche positions and breadths of all sampled beetle species in relation to deadwood exposure time. Points represent the abundance-weighted mean year of emergence for each beetle species, calculated by averaging all emergence years weighted by the number of individuals per year. Vertical lines represent standard deviation around the mean, reflecting the temporal spread of emergence (interpreted as niche breadth). Tree species associations (i.e., from which tree species the beetle species emerged) are indicated by abbreviations on the right (B = beech, F = fir, S = spruce). The horizontal grey dashed lines indicate the unique beetle compositions identified by the conditional inference tree for years 1-2, 3, 4 and 5-12.

# **Section 8: Venn diagram**

**
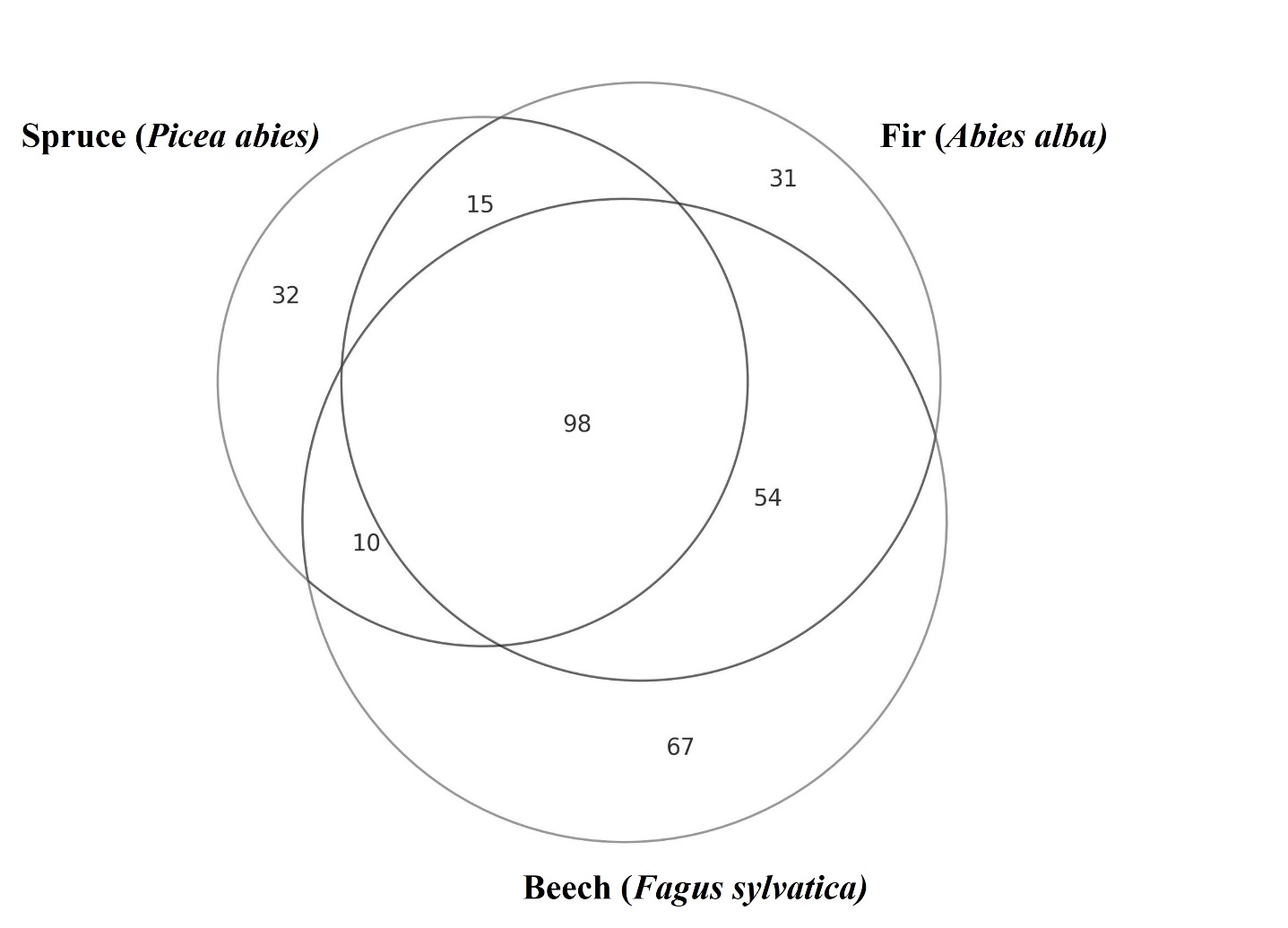
Figure S8:** Venn diagram of saproxylic beetle species collected over 12 years from spruce, fir, and beech logs. Numbers represent species unique to each tree species or shared between them. A total of 297 species were recorded, with 98 species occurring in all three tree species.

# **References**

Cadotte, M., Albert, C. H., & Walker, S. C. (2013). The ecology of differences: Assessing community assembly with trait and evolutionary distances. *Ecology Letters*, *16*(10), 1234–1244. https://doi.org/10.1111/ele.12161

Cadotte, M., & Davies, T. J. (2016). *Phylogenies in ecology: A guide to concepts and methods*. Princeton University Press.

Gossner, M. M., Lachat, T., Brunet, J., Isacsson, G., Bouget, C., Brustel, H., Brandl, R., Weisser, W. W., & Müller, J. (2013). Current near‐to‐nature forest management effects on functional trait composition of saproxylic beetles in beech forests. *Conservation Biology*, *27*(3), 605–614.

Hagge, J., Müller, J., Birkemoe, T., Buse, J., Christensen, R. H. B., Gossner, M. M., Gruppe, A., Heibl, C., Jarzabek‐Müller, A., Seibold, S., Siitonen, J., Soutinho, J. G., Sverdrup‐Thygeson, A., Thorn, S., & Drag, L. (2021). What does a threatened saproxylic beetle look like? Modelling extinction risk using a new morphological trait database. *Journal of Animal Ecology*, *90*(8), 1934–1947. https://doi.org/10.1111/1365-2656.13512

Hartig, F. (2022). *DHARMa: Residual diagnostics for hierarchical (multi-level/mixed) regression models* (Version 0.4.6) [Computer software]. 10.32614/CRAN.package.DHARMa

Hunt, T., Bergsten, J., Levkanicova, Z., Papadopoulou, A., John, O. St., Wild, R., Hammond, P. M., Ahrens, D., Balke, M., Caterino, M. S., Gómez-Zurita, J., Ribera, I., Barraclough, T. G., Bocakova, M., Bocak, L., & Vogler, A. P. (2007). A Comprehensive Phylogeny of Beetles Reveals the Evolutionary Origins of a Superradiation. *Science*, *318*(5858), 1913–1916. https://doi.org/10.1126/science.1146954

Kembel, S. W., Cowan, P. D., Helmus, M. R., Cornwell, W. K., Morlon, H., Ackerly, D. D., Blomberg, S. P., & Webb, C. O. (2010). Picante: R tools for integrating phylogenies and ecology. *Bioinformatics*, *26*(11), 1463–1464. https://doi.org/10.1093/bioinformatics/btq166

Köhler, F. (2000). *Totholzkäfer in Naturwaldzellen des nördlichen Rheinlands: Vergleichende Studien zur Totholzkäferfauna Deutschlands und deutschen Naturwaldforschung*. LÖBF, Landesamt für Agrarordnung Nordrhein-Westfalen.

Maechler, M., Rousseeuw, P., Struyf, A., Hubert, M., & Hornik, K. (2022). cluster*: Cluster Analysis Basics and Extensions* (Version R package version 2.1.4) [Computer software]. https://CRAN.R-project.org/package=cluster

Müller, J., Noss, R. F., Bussler, H., & Brandl, R. (2010). Learning from a “benign neglect strategy” in a national park: Response of saproxylic beetles to dead wood accumulation. *Biological Conservation*, *143*(11), 2559–2569. https://doi.org/10.1016/j.biocon.2010.06.024

Neff, F., Hagge, J., Achury, R., Ambarlı, D., Ammer, C., Schall, P., Seibold, S., Staab, M., Weisser, W. W., & Gossner, M. M. (2022). Hierarchical trait filtering at different spatial scales determines beetle assemblages in deadwood. *Functional Ecology*, *36*(12), 2929–2942. https://doi.org/10.1111/1365-2435.14186

Polilov, A. A., Ribera, I., Yavorskaya, M. I., Cardoso, A., Grebennikov, V. V., & Beutel, R. G. (2019). The phylogeny of Ptiliidae (Coleoptera: Staphylinoidea) – the smallest beetles and their evolutionary transformations. *Arthropod Systematics & Phylogeny*, *77*(3), 433–455. https://doi.org/10.26049/ASP77-3-2019-4

R Core Team. (2023). *R: A Language and Environment for Statistical Computing* [Computer software]. https://www.R-project.org/

Seibold, S., Bässler, C., Brandl, R., Büche, B., Szallies, A., Thorn, S., Ulyshen, M. D., & Müller, J. (2016). Microclimate and habitat heterogeneity as the major drivers of beetle diversity in dead wood. *Journal of Applied Ecology*, *53*(3), 934–943. https://doi.org/10.1111/1365-2664.12607

Seibold, S., Brandl, R., Buse, J., Hothorn, T., Schmidl, J., Thorn, S., & Müller, J. (2015). Association of extinction risk of saproxylic beetles with ecological degradation of forests in Europe. *Conservation Biology*, *29*(2), 382–390. https://doi.org/10.1111/cobi.12427

Thorn, S., Bässler, C., Bußler, H., Lindenmayer, D. B., Schmidt, S., Seibold, S., Wende, B., & Müller, J. (2016). Bark-scratching of storm-felled trees preserves biodiversity at lower economic costs compared to debarking. *Forest Ecology and Management*, *364*, 10–16. https://doi.org/10.1016/j.foreco.2015.12.044

Yu, G., Smith, D. K., Zhu, H., Guan, Y., & Lam, T. T. (2017). ggtree: An r package for visualization and annotation of phylogenetic trees with their covariates and other associated data. *Methods in Ecology and Evolution*, *8*(1), 28–36. https://doi.org/10.1111/2041-210X.12628
